# Receptor-Interacting Protein Kinase 3-Mediated Modulation of Endothelial Cell Necroptosis and Mitochondrial Dysfunction through AMPK/Drp1 Signaling Pathway: Insights into the Pathophysiological Mechanisms of Lipopolysaccharide-Induced Acute Lung Injury

**DOI:** 10.7150/ijms.104932

**Published:** 2025-01-01

**Authors:** Zhaoning Zhao, Pingjun Zhu, Yue Lou, Ruoyu Hou, Heqiang Sun, Yingzhen Du, Guogang Xu

**Affiliations:** 1 Health Management Institute, The Second Medical Center & National Clinical Research Center for Geriatric Diseases, Chinese PLA General Hospital, Beijing 100853, China.; 2 Chinese PLA General Hospital, Medical School of Chinese PLA, Beijing 100853, China.; 3 Department of Respiratory and Critical Care Medicine, The Second Medical Center & National Clinical Research Center for Geriatric Diseases, Chinese PLA General Hospital, Beijing 100853, China.; 4 The Second Medical Center, Chinese PLA General Hospital, Beijing 100853, China.; 5 School of Biology, University of St Andrews, St Andrews, KY16 9ST, UK.; 6 Department of Laboratory Medicine, The Second Medical Center & National Clinical Research Center for Geriatric Diseases, Chinese PLA General Hospital, Beijing 100853, China.; 7 Department of Disease Control and Prevention, The Second Medical Center & National Clinical Research Center for Geriatric Diseases, Chinese PLA General Hospital, Beijing 100853, China.

**Keywords:** Ripk3, Acute lung injury, Cell necroptosis, Mitochondrial damage

## Abstract

Receptor-interacting protein 3 (Ripk3) plays a crucial part in acute lung injury (ALI) by regulating inflammation-induced endothelial damage in the lung tissue. The precise mechanisms through which Ripk3 contributes to the endothelial injury in ALI still remain uncertain. In the current research, we employed Ripk3-deficient (Ripk3^-/-^) mice to examine the role of Ripk3 in ALI progression, focusing on its effects on endothelial cells (ECs), mitochondrial damage and necroptosis. Our study observed significant Ripk3 upregulation in lipopolysaccharide- (LPS-) treated lung tissues, as well as in murine pulmonary microvascular endothelial cells (PMVECs). Ripk3 deletion improved lung tissue morphology, reduced inflammation, oxidative stress and endothelial dysfunction under LPS challenge. It also mitigated LPS-induced necroptosis and mitochondrial damage in PMVECs. Ripk3 upregulation suppressed the AMP-activated protein kinase (AMPK) pathway and activated Drp1-mediated mitochondrial fission, increasing mitochondrial permeability transition pore (mPTP) opening and PMVEC necroptosis. Conversely, Ripk3 deletion activated the AMPK/Drp1-mitochondrial fission pathway, preventing mPTP opening and PMVEC necroptosis in ALI. These findings demonstrated that Ripk3 promotes necroptosis through the AMPK/Drp1/mPTP opening pathway, identifying a potential therapeutic target for ALI treatment.

## Introduction

Acute respiratory distress syndrome (ARDS) is a widespread lung injury that arises from several pathological stresses such as ischemia, anoxia, severe infection, and trauma 1, 2. ARDS is defined by an exaggerated inflammatory response that leads to the breakdown of the alveolar-capillary barrier (ACB) and fluid accumulation in the lungs, causing pulmonary edema 3, 4. It is further characterized by persistent hypoxemia, reduced pulmonary compliance, and the presence of abnormal shadows on chest imaging 5, considered to be a prevalent refractory complication with a substantial mortality rate (30-40%) 6 among severely ill patients 7, 8. Currently, there is a dearth of effective therapy for ARDS. Based on the pathogenic mechanism of ARDS, disseminating stimulation of inflammation influences pulmonary microvascular endothelium first, which leads to an increase in its permeability to an abnormal state 9, 10. As the major component of ACB, pulmonary microvascular endothelium typically maintains its permeability at an appropriate level to serve its function 11. This hyperpermeability of pulmonary microvascular endothelial cells (PMVECs) harms the integrity of ACB and allows the fluid to undergo osmosis from capillary to alveoli. This leakage brings inflammatory cells to alveolar space, and leads to edema in lung tissues eventually 12. Thus, it is crucial to discover practical and effective strategies to enhance the impairment of barrier function in PMVECs for treating ARDS.

Mitochondria, acting as the cellular powerhouse, regulate various physiological processes, including signaling transmission, oxidative stress, cell growth and death 13, particularly in some severe conditions, for instance ARDS. The involvement of mitochondrial-derived reactive oxygen species (ROS) in ARDS has garnered significant interest due to its regulatory function 14. Excessive ROS have a permanent detrimental impact on endothelial cells 15. Moreover, oxidative stress in endothelial cells intensifies apoptosis, promotes disruption of the protective barrier, thus causes damage to the lungs 16. In cases of lipopolysaccharide- (LPS-) induced acute lung injury (ALI), there is a reduction in the production of mitochondrial adenosine triphosphate (ATP), while ROS levels increase due to the concentration of neutrophils in the lung tissues 17. These data suggest that mitochondrial dysfunction plays a part in ALI, while the specific mechanisms remain incompletely understood. The morphology of mitochondria is sustained by the dynamic and contrasting actions of mitochondrial fusion and fission 18. Mitochondrial fusion facilitates the dispersion of harmful substances, while mitochondrial fission permits the separation and eventual breakdown of damaged and depolarized mitochondria. An imbalance in the process of mitochondrial fission and fusion has been associated with a diverse range of disorders 19. Recent data have indicated that changes in mitochondrial morphology are preceded by mitochondrial damage 20, 21. Augmented mitochondrial fragmentation serves as an initial indication of mitochondrial membrane potential (MMP) depolarization and overproduction of mitochondrial ROS. Although the significant contribution of mitochondrial fusion to ARDS is widely acknowledged, there is still limited knowledge on alteration of mitochondrial homeostasis in ARDS progression.

ARDS may involve several forms of cellular demise, such as autophagy, apoptosis, and necroptosis 22. Necroptosis, in particular, enhances inflammation and has a significant impact on ARDS. Receptor-interacting protein 3 (Ripk3) takes control of necroptosis, a programmed cell death that has been recently identified 23. Necroptosis depends on the phosphorylation of Ripk3, which acts on mixed lineage kinase domain-like pseudokinase (MLKL) as its substrate 24. Necroptosis is thought to play a part in endothelial dysfunction 25, involving the progressive stimulation of the inherent immune response and blood clotting, which enhances endothelial cell permeability 26. Nevertheless, the precise mechanism by which necroptosis occurs in ARDS remains unidentified. Several studies demonstrate that lung injury in severe ARDS triggered by large doses of LPS is mainly caused by Ripk3-MLKL-mediated necroptosis and endothelial dysfunction, suggesting its potential role in the development of ARDS 26, 27. In addition, some studies have observed that Ripk3 has a crucial impact on the occurrence of mitochondrial dysfunction, including mitochondrial fission and mitochondrial oxidative stress in pathological conditions, for instance ALI 28. In light of the correlation between necroptosis and mitochondrial injury, our objective is to investigate whether the initiation of necroptosis results from changes in mitochondrial morphology and consequently causes lung injury associated with ARDS. We hypothesize that Ripk3 influences mitochondrial fission, leading to the initiation of necroptosis in the development of ARDS.

## Materials and Methods

### Animal model

The laboratory animals at this facility were handled in accordance with the Guidelines for the Care and Use of Laboratory Animals. Ethics approval was granted by the Chinese PLA General Hospital Ethics Committee in Beijing, China. The C57BL/6 mice used in this study were genetically modified to be Ripk3-deficient (Ripk3^-/-^). The ages and sexes of all experimental mice were matched. LPS (5 mg/kg) was injected intratracheally for 24 hours to induce ALI in the animal model 29. The control group (Sham) received injections of phosphate-buffered saline (PBS) and was observed for 24 hours. The bronchoalveolar lavage fluid (BALF) and lung tissue were harvested for subsequent assessment.

### Analysis of histology

Preserved lung tissues were embedded in paraffin blocks after being treated with a 4% fixative solution (Sigma-Aldrich). Each specimen was sliced into 5μm thick, then stained with hematoxylin and eosin (H&E, Sigma-Aldrich). The areas of interest were analyzed with Image J software.

### Lung wet to dry ratio (W/D)

Edema severity was determined by calculating the W/D ratio. The lung tissues were measured in terms of weight following the removal of the left lung and the extraction of blood. After 4 days of drying at 80°C, the specimen reached a constant weight.

### Evens Blue assay

Evans Blue dye (EB, 35 mg/kg, Sigma-Aldrich) was administered to the mice intraperitoneally two hours before euthanasia. Following this, the pulmonary circulation was flushed with normal saline for 2 minutes to remove any remaining EB in the blood vessels. Then, the lungs were removed, homogenized in 1 mL PBS, and incubated in 2 mL formalin at 60°C for 24 hours. The supernatants containing the extracted EB dye were collected, and the absorbance of the resulting supernatant was measured at 620 nm to quantify the concentration of EB dye.

### Analysis of protein content and cell count in BALF

Murine BALF was obtained by injecting PBS intratracheally after mice euthanization. The Bradford Protein Quantification Kit (Beyotime, China) was applied to quantifying the protein concentration in BALF following centrifugation at 1500 rpm at 4°C for 10 minutes. A hemocytometer and Wright-Giemsa staining (Beyotime, China) were conducted to quantify the total cell count and several types of hemocytes, such as macrophages, neutrophils, and leukocytes 30. The levels of proinflammatory cytokines (tumor necrosis factor-alpha (TNF-α), interleukin-1β (IL-1β), interleukin-6 (IL-6), and monocyte chemoattractant protein-1 (MCP-1)) were estimated with ELISA kits (R&D, USA).

### Cell culture and treatment

The mouse pulmonary microvascular endothelial cell line was acquired from Shanghai Univ, China. DMEM (Gibco, USA) was used to culture PMVECs added with 10% fetal bovine serum (FBS, Gibco, USA). The cells were cultivated in a humidified incubator maintained at 37°C with 5% carbon dioxide. The PMVECs were incubated under LPS (10 μg/ml, Sigma-Aldrich, USA) for 12 h to cause ALI 31.

### Gene-specific knockdown

A lentivirus system (OBiO, Shanghai, China) was employed to suppress the expression level of Ripk3, following the manufacturer's protocol. A total of 10^5^ cells each well were cultured in 12-well plates and infected with lentiviral shRNA at 50 multiplicity of infection (MOI) for 24 h. Subsequently, cells were grown in DMEM containing 10% FBS for three days. Nonsilencing scrambled short hairpin RNA (shRNA) free of EGFP served as the negative control. The transfected cells were subjected to LPS treatment as previously mentioned, and then collected for further analysis.

### Cell necroptosis

Necroptosis was identified by flow cytometry with a kit (Vazyme Biotech). Following treatment, the cells were gathered, rinsed with PBS, then resuspended in 100μL binding buffer containing 5μL Annexin V-FITC and PI, and incubated at ambient temperature for 10 minutes. After incubation, flow cytometry (Thermo Fisher Scientific, USA) was employed to identify necroptosis.

### Western blotting

4-12% sodium dodecyl sulfate-polyacrylamide gels (SDS-PAGE) were applied for protein samples (25-35μg). Then polyvinylidene fluoride (PVDF) membrane (EMD Millipore) covered the SDS-PAGE. Reference was taken from the pertained protein ladder (Thermo Fisher Scientific, 26616). 5% skim milk submerged the membranes later. After the 1 hour coverage, primary antibodies and secondary antibodies were added, incubated overnight under 4°C and for 1 hour at RT respectively. TBST was required to wash membranes after two incubations. The signals were detected using ECL Detection Reagent (GE Healthcare). The luminosity of the bands was analyzed with LAS 400 system (GE Healthcare), and normalized to GAPDH.

### Transmission electron microscopy (TEM)

Following LPS treatment, a 2-hour fixation process on the samples was conducted initially with 2.5% glutaraldehyde at 4°C, followed by post-fixation in 1 % osmium tetroxide at RT for an additional 2 hours. Subsequently, the samples underwent dehydration in a serial diluted ethanol group (65 %, 70 %, 75 %, 80 %, and 95 % ethanol for 10 minutes each) before being placed in epoxy resin. Afterward, uranyl acetate and lead citrate (Ted Pella, USA) were used to stain the ultrathin section (70nm). Finally, images were visualized utilizing a TEM microscope (JEM1400, Japan).

### Isolation of mitochondrial and cytosolic fractions

Fractions of mitochondria and cytosol were isolated using a kit (Beyotime) as described by the protocol of manufacturer. Cells were collected and resuspended in an isolation buffer with 1mM PMSF, then left to incubate for 10 minutes on ice. This suspension was homogenized and centrifuged at 1000 × g at 4°C for 10 minutes. Next, the supernatant was gathered and subjected to centrifugation at 11000 × g at 4°C for 10 minutes. The mitochondrial and cytosolic fractions were distinguished by resuspending the deposit in the mitochondrial lysis buffer after separating the supernatant.

### Analyses of mitochondrial membrane potential and mitochondrial permeability transition pore (mPTP) opening

JC-1 probes (Beyotime) were used to measure mitochondrial membrane potential. The fluorescence intensity of tetramethylrhodamine ethyl ester (TMRE) reflects the haphazard opening rate of mPTP 32. As a negative control, Cyclosporin A (CsA, 10μM, Sigma Aldrich) was used to inhibit the opening of mPTP.

### ROS, glutathione (GSH), superoxide dismutase (SOD), and malondialdehyde (MDA) assays

GSH level, SOD activity, and MDA level were assessed following the manufacturer's guidelines. Previously reported technique such as 2,7′-dichlorofluorescein-diacetate (DCFHDA, Beyotime, China) staining 33 was used to detect ROS.

### Single-cell RNA sequencing (scRNA-seq) analysis

The samples were integrated by anchors method from the R package "Seurat" 34, and core cells were acquired by filtering scRNA-seq. The first step involved the removal of low-quality data through three methods: filtering out single cells with less than 5 genes expressed, eliminating cells expressing less than 100 genes, and excluding cells with more than 5% mitochondrial genes. Subsequently, gene expression of core cells was normalized using a linear regression model, and then the top 2000 genes with highly variable characteristics were screened by analysis of variance (ANOVA). Principal component analysis (PCA) was performed on single-cell samples, and the top 20 principal components (PC) were selected for subsequent analysis. Cell clustering analysis was performed using the "FindNeighbors" and "FindClusters" functions, and T-distributed random neighborhood embedding (t-SNE) was carried out through the "RunTSNE" function for visualization. Gene expression differences in different samples were assessed utilizing the Wilcoxon-Mann-Whitney test. Using the "singleR" package from R 35, three databases including HumanPrimaryCellAtlasData, BlueprintEncodeData, and ImmuneCellExpressionData were utilized as the reference for auxiliary annotation, followed by the CellMarker database 36 and previous studies to find marker genes for manual annotation of different clusters.

### Analyses of statistics

The involvement of unpaired t-test aimed to construct a comparison between two clusters. The one-way ANOVA was applied to compare discrepancies between at least three independent experiments. In all cases, results were demonstrated in the form of mean ± standard deviation (SD), and the significance was set at *P* < 0.05.

## Results

### Ripk3 is increased in lung ECs following LPS challenge

**Figure 1:**
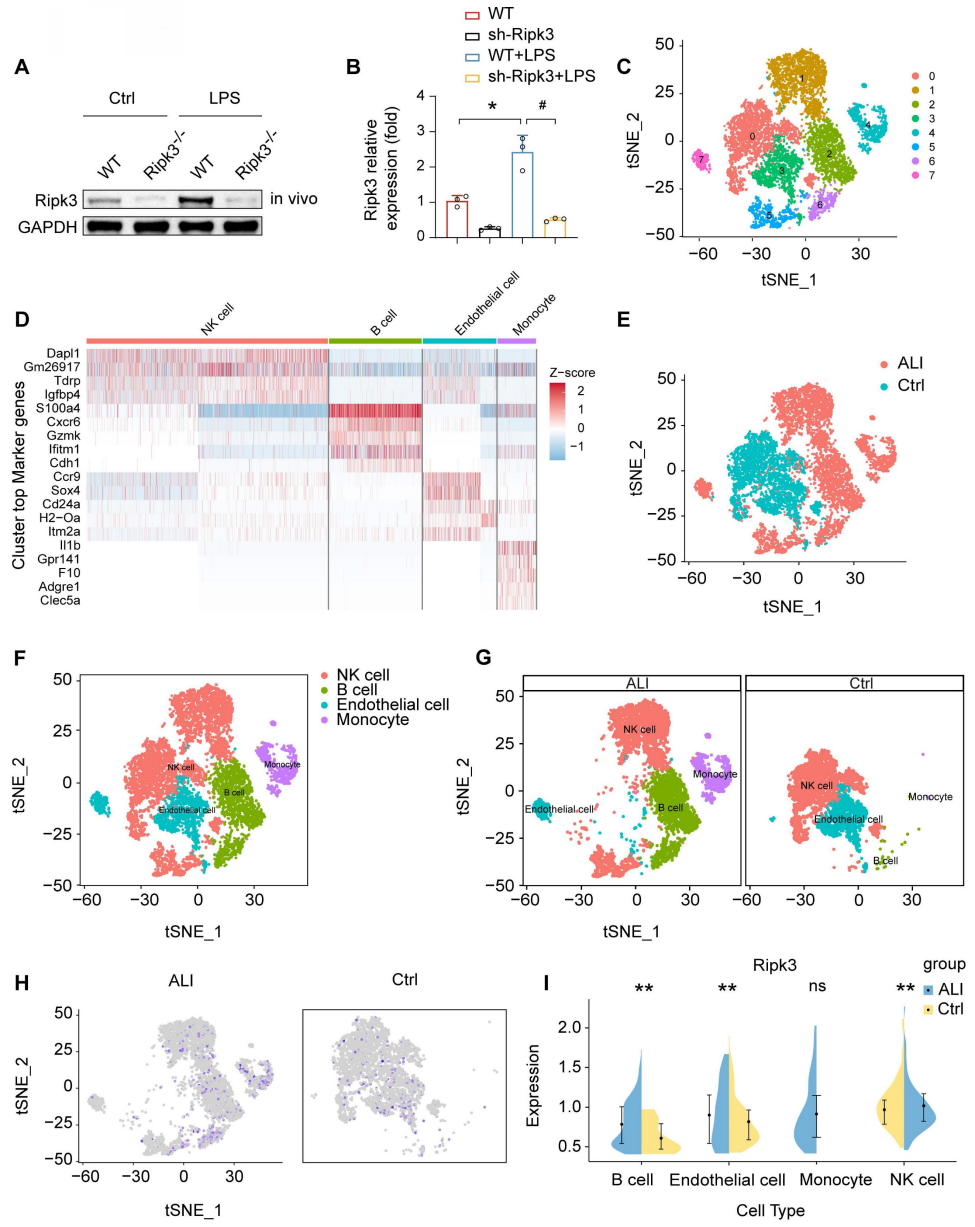
** LPS upregulates Ripk3 in lung ECs.** (A**-**B) The change of Ripk3 expression *in vivo* was quantified using western blotting. (C-I) Ripk3 expression in different cell types from the lung was determined using single-cell sequencing analysis. Mean ± SEM, ∗*p* < 0.05 vs. the wild-type (WT) group; #*p* < 0.05 vs. the LPS group.

Analysis of Ripk3 expression in lung tissues was conducted using Western blotting. The findings revealed an upregulation of Ripk3 in lung tissues after LPS treatment compared to baseline levels **(Fig. 1A-B)**. To determine the main cellular source of Ripk3 in lung tissue, we performed single-cell analyses using the Gene Expression Omnibus (GEO) database, which contains single-cell profiles from mouse lung samples, with and without septic stress. The results indicated a high abundance of Ripk3 in ECs **(Fig. 1C-I)**. Suggestion could be drawn that Ripk3 is highly involved during acute lung injury pathogenesis, meriting further exploration as a potential therapeutic target.

### Ripk3 disrupts the pulmonary vascular endothelial barrier in ALI mice

**Figure 2:**
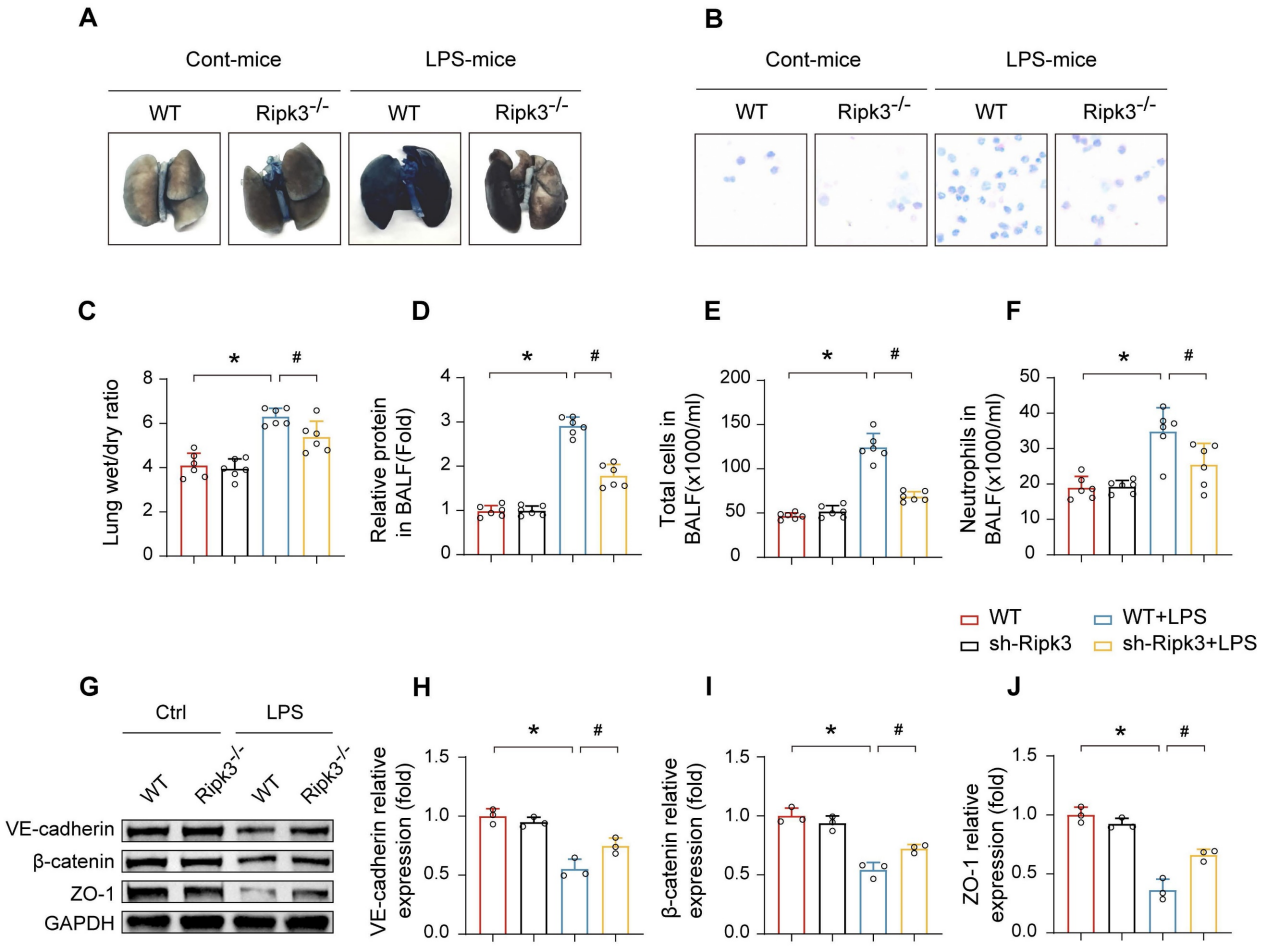
** Effect of Ripk3 deletion on the alveolar-capillary barrier in LPS-induced ALI mice.** (A) Representative images of Evans Blue (EB) extravasation in the lungs. (B-F) The ratio of wet weight to dry weight of the lungs, the protein content, the number of total cells and neutrophils in BALF were analyzed to assess lung permeability. (G-J) The protein levels of VE-cadherin, β-catenin, and ZO-1 in the lung tissues were measured using western blot and quantitative analysis. Mean ± SEM, ∗*p* < 0.05 vs. the WT group; #*p* < 0.05 vs. the LPS group.

To investigate the relationship between elevated Ripk3 expression and LPS-induced lung injury, Ripk3^-/-^ mice were employed. The results demonstrated that LPS exposure led to augmented pulmonary vascular permeability, as evidenced by significant increases in Evans Blue extravasation, lung W/D weight ratio, BALF protein concentration, total cell count, and neutrophils in the BALF **(Fig. 2A-F)**. To assess microvascular barrier function, the expressions of key intercellular junction proteins in lung tissues including VE-cadherin, β-catenin, and ZO-1, were examined by Western blot analysis, which revealed a marked downregulation following LPS administration. However, these alternations were apparently reversed in Ripk3 knockout mice **(Fig. 2G-J)**. These results suggest that Ripk3 participates intensively in compromising pulmonary vascular endothelial barrier integrity during ALI.

### Ripk3 deletion reduces LPS-induced pulmonary pathological damage, inflammation, and oxidative injury

**Figure 3:**
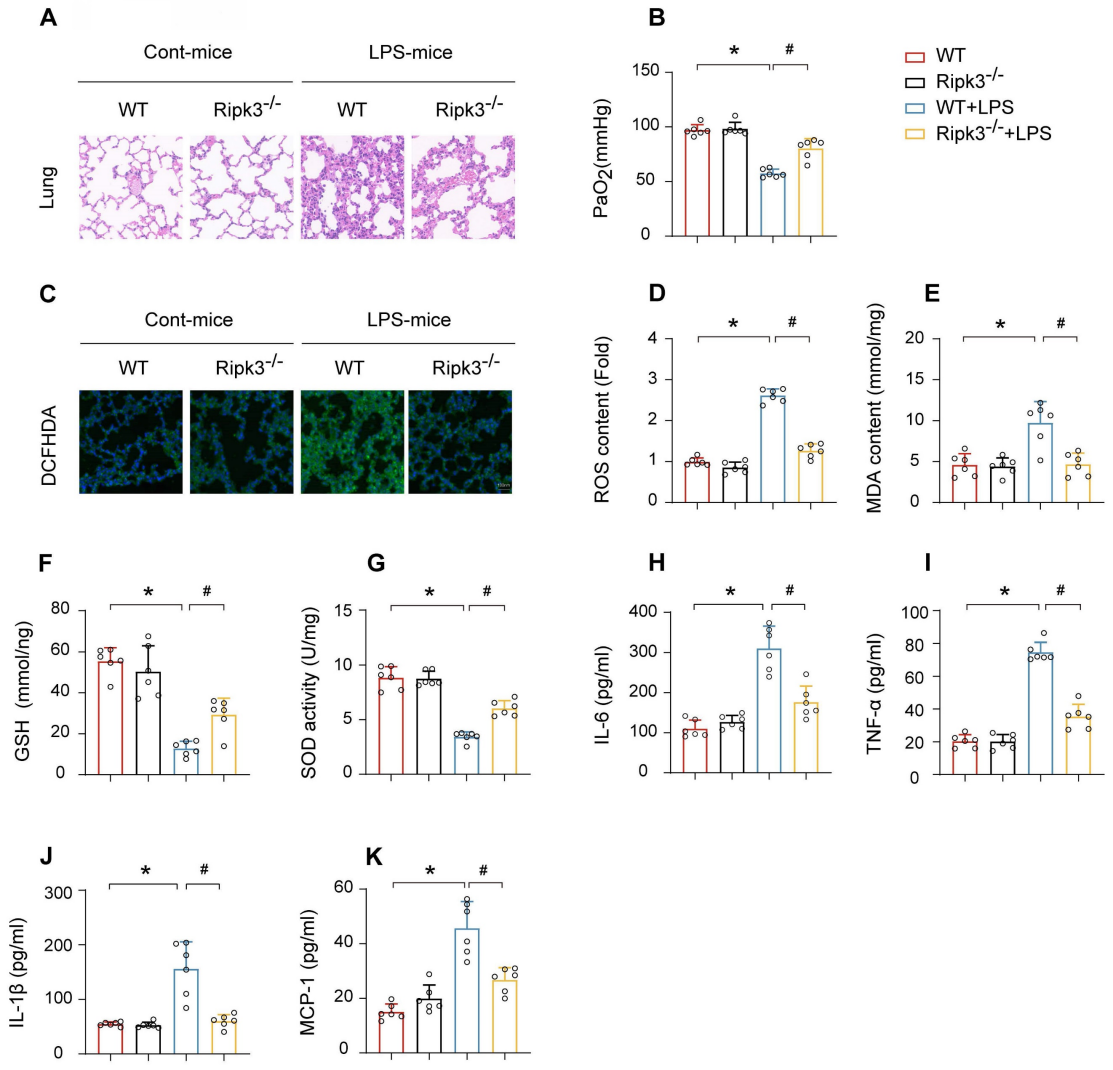
** Ripk3 deletion attenuates LPS-mediated oxidative stress and inflammation response in acute lung injury.** (A) Pathological alterations of lung parenchyma observed by HE staining after acute lung injury. (B) Measurement of the partial pressure of arterial oxygen (PaO_2_). (C-D) DCFHDA staining was used to detect ROS content. (E-G) MDA level, SOD activity, GSH level were also measured in lung tissue. (H-K) Protein levels of IL-6, TNF-α, IL-1β, and MCP-1 were quantified by ELISA. Mean ± SEM, ∗*p* < 0.05 vs. the WT group; #*p* < 0.05 vs. the LPS group.

HE staining of lung tissues from mice treated with LPS revealed significant pathological alterations, including inflammatory cell infiltration, interstitial and alveolar edema, hemorrhage, and diffuse alveolar damage. The alterations were notably attenuated in Ripk3-deficient mice **(Fig. 3A)**. Furthermore, Ripk3 ablation improved the partial pressure of arterial oxygen (PaO_2_) in response to LPS challenge **(Fig. 3B)**. Improvement in PaO2 accelerated the repair process of pulmonary vascular endothelial cells and improved respiratory function while reducing the risk of multiple organ dysfunction syndrome (MODS). Given the important role of oxidative stress in ALI, we investigated whether Ripk3 deletion protects against oxidative injury. DCFHDA staining demonstrated elevated ROS levels in the lung tissue of mice after LPS challenge, which reduced to baseline levels in Ripk3-deficient mice **(Fig. 3C-D)**. Additionally, LPS stimulation increased MDA production and decreased the levels of antioxidant factors such as GSH and SOD. Ripk3 deletion normalized these changes **(Fig. 3E-G)**. LPS induced elevated expression of inflammatory cytokines (IL-6, TNF-α, IL-1β, and MCP-1) in lung tissues. Ripk3 deletion demonstrated anti-inflammatory effects by lowering these cytokine levels following LPS exposure **(Fig. 3H-K)**. In conclusion, Ripk3 deletion protects against LPS-induced ALI by alleviating inflammation, oxidative stress, and tissue damage.

### Ripk3 evokes lung ECs necroptosis via promoting mPTP opening

**Figure 4:**
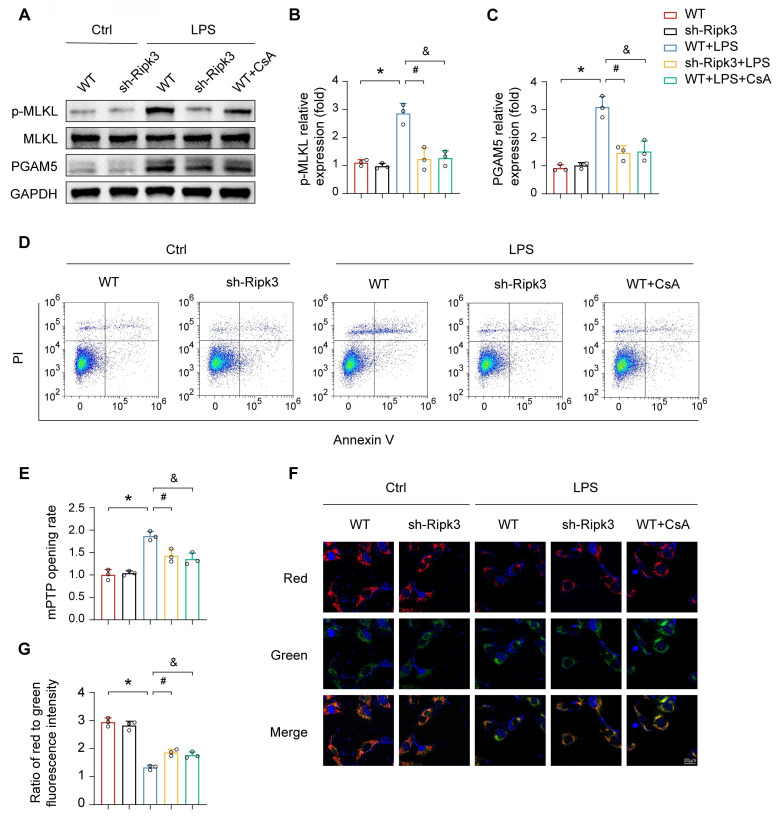
** Ripk3 knockdown reduces PMVEC necroptosis by inhibiting mPTP opening.** (A-C) Western blots were used to analyze the expression of PGAM5, and p-MLKL. (D) Necroptosis of PMVECs was quantified by flow cytometry with Annexin V/PI staining. The necroptosis group: the percentage of PI^+^ cells. Cyclosporin A (CsA) was used as the inhibitor to prevent mitochondrial permeability transition pore (mPTP) opening. (E) Evaluation of the rate of mPTP opening. (F-G) Changes of mitochondrial membrane potential were identified using JC-1 staining. Mean ± SEM, ∗*p* < 0.05 vs. the Ctrl group; #*p* < 0.05 vs. the LPS group; &*p* < 0.05 vs. the LPS+CsA group.

ALI is characterized by the loss of functional cells due to cell death. To explore the potential protective effect of Ripk3 deletion against LPS-triggered vascular endothelial cell necroptosis, a series of experiments were conducted. Deficiency of Ripk3 was found to attenuate cellular necroptosis in lung tissue caused by LPS, as evidenced by reduced levels of phosphoglycerate mutase 5 (PGAM5) and phosphorylated mixed lineage kinase domain-like protein (p-MLKL). Cellular experiments further supported the protective role of Ripk3 deficiency in acute lung injury. In PMVECs, LPS exposure increased the necroptosis index, as indicated by raised PGAM5 and p-MLKL levels. Nevertheless, this effect was nullified in Ripk3 knockdown cells **(Fig. 4A-C)**. Annexin V/PI staining showed that LPS increased necroptosis in PMVECs, which was significantly reduced by Ripk3 inhibition **(Fig. 4D)**. These findings collectively suggest that LPS stimulates the activation of necroptosis in PMVECs by increasing Ripk3 expression.

To clarify the mechanism of Ripk3-mediated necroptosis, the study paid attention to mPTP opening, which has been recognized as an upstream trigger of cellular necroptosis. As shown in **Fig. 4E**, LPS significantly prolonged mPTP opening time, an effect that was attenuated in Ripk3-deficient cells. Mitochondrial membrane potential, which typically dissipates upon mPTP opening, showed similar trends. CsA, an mPTP opening inhibitor, was used as a negative control. CsA increased cellular viability under LPS stress, similar to the protective effect of Ripk3 inhibition **(Fig. 4F-G)**. These findings suggest that Ripk3-initiated necroptosis occurs through mPTP opening in ECs.

### Ripk3 facilitates mPTP opening via Drp1-related mitochondrial fission

**Figure 5:**
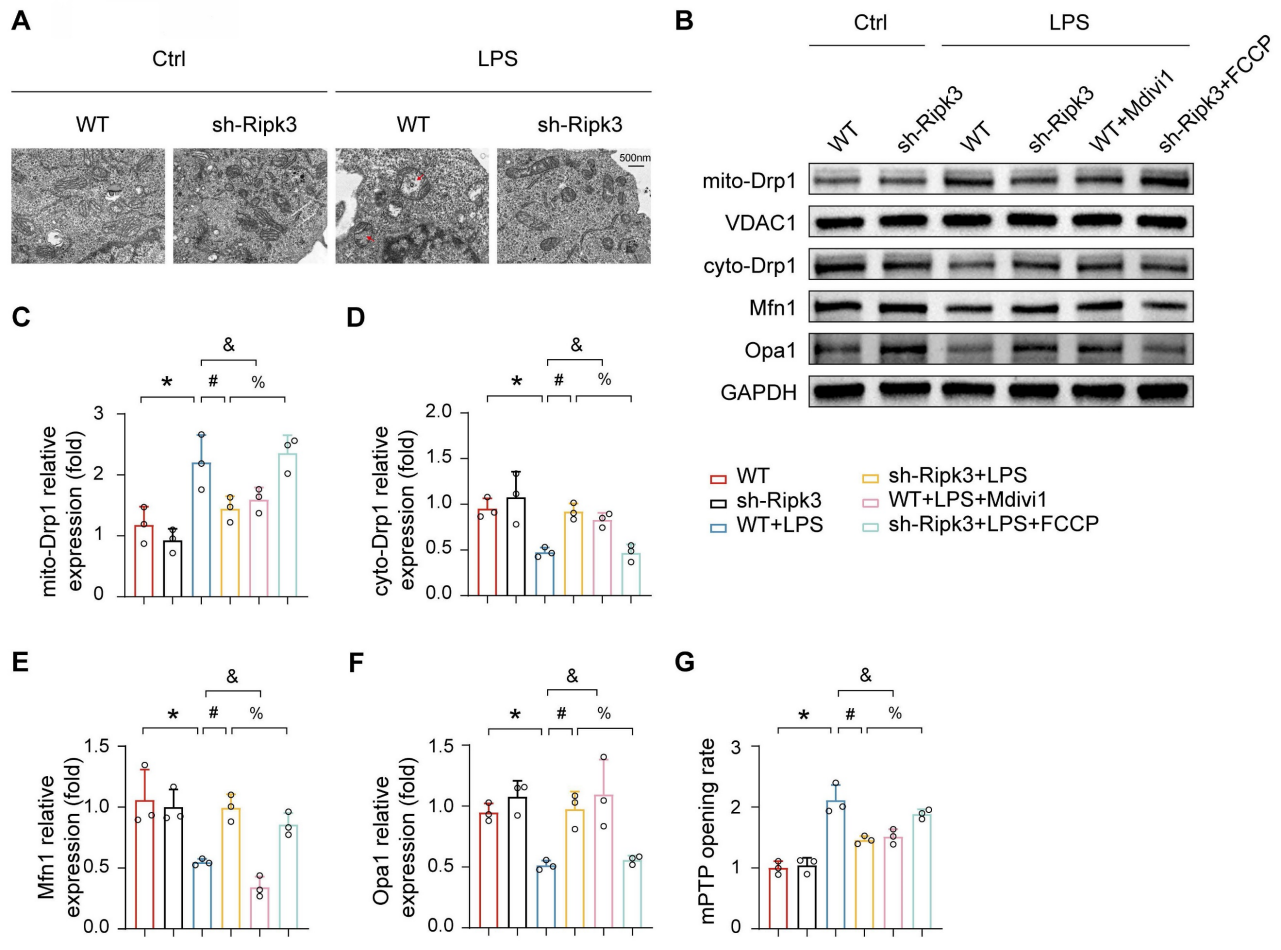
** Ripk3 knockdown inhibits mPTP opening via Drp1-related mitochondrial fission.** (A) Representative transmission electron microscopy (TEM) images depicting changes in mitochondrial morphology. Red arrow: damaged and swollen mitochondria. (B-F) The expression levels of mito-Drp1, cyto-Drp1, Mfn1, and Opa1 were quantified by Western blotting. The mitochondrial fission inhibitor, Mdivi1, was utilized to impede mitochondrial fission in WT cells under LPS administration. The mitochondrial fission activator, FCCP, was employed to promote mitochondrial fission in Ripk3-deficient cells under LPS treatment. (G) The change of mPTP opening time. Mean ± SEM, ∗*p* < 0.05 vs. the Ctrl group; #*p* < 0.05 vs. the LPS group; &*p* < 0.05 vs. the LPS+Mdivi1 group; %*p* < 0.05 vs. the LPS+ FCCP group.

Previous research has demonstrated that Ripk3 deficiency helps maintain mitochondrial balance by normalizing mitochondrial dynamics, including fission and mitophagy. Additionally, enhanced mitochondrial fission has been linked to mPTP opening. Based on these observations, we investigated whether Ripk3 induces mPTP opening by promoting mitochondrial fission. TEM imaging revealed that LPS treatment caused significant alterations in mitochondrial morphology, which were characterized by partial mitochondrial fragmentation, loss of cristae, vacuole formation, and irregular arrangement **(Fig. 5A)**. Ripk3 knockdown cells showed reversal of LPS-induced mitochondrial abnormalities. Western blot analysis revealed that LPS increased mitochondrial Drp1 expression, while decreasing cytoplasmic Drp1 expression. **(Fig. 5B-D)**. Interestingly, Ripk3 inhibition reversed this phenomenon. Furthermore, Ripk3 deficiency resulted in elevated expression levels of mitofusin-1 (Mfn1) and optic atrophy 1 (Opa1), both of which are involved in mitochondrial fusion **(Fig. 5B, E-F)**. These findings suggest that LPS excessively stimulates mitochondrial fission, possibly due to increased Ripk3 expression. To assess whether Ripk3 knockdown protects against mPTP opening in LPS-treated PMVECs by inhibiting mitochondrial fission, we used the fission inhibitor Mdivi1 (20μM). Conversely, we treated Ripk3-deficient cells with the fission activator FCCP (5μM) under LPS conditions **(Fig. 5G)**. The results confirm that LPS and FCCP triggered mPTP opening, while Ripk3 knockdown and Mdivi1 inhibited it. These results indicate that Ripk3 induces mPTP opening through Drp1-mediated mitochondrial fission.

### Ripk3 regulates Drp1 activation via the AMPK pathway

**Figure 6:**
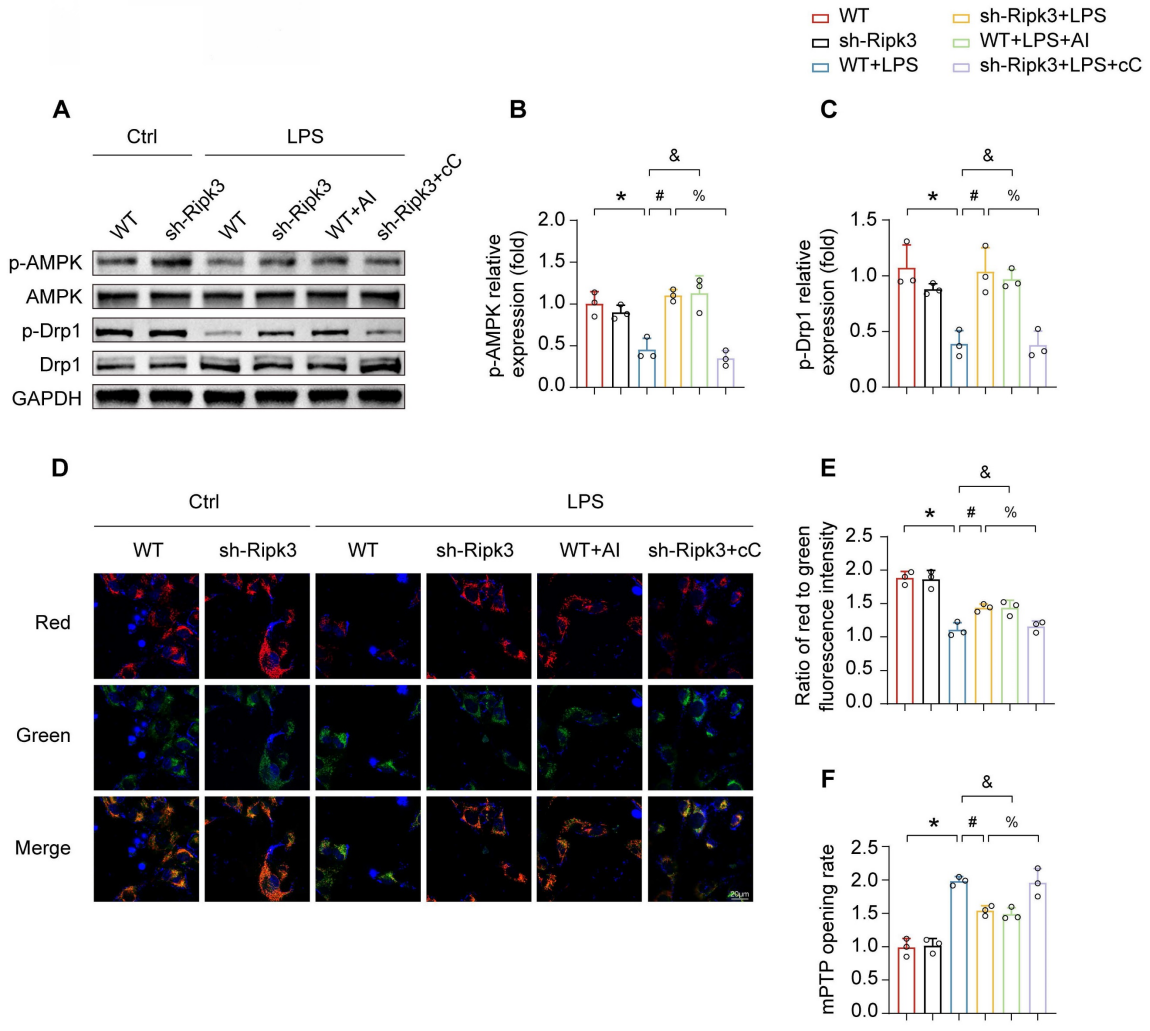
** Ripk3 knockdown alleviates mitochondrial injury through promoting AMPK-mediated Drp1 phosphorylation.** (A-C) The phosphorylation of Drp1 and AMPK was measured using western blots. The AMPK pathway activator, AICAR (AI), was employed to activate AMPK pathway in WT cells under LPS treatment. The AMPK pathway inhibitor, compound C (cC), was utilized to block the AMPK pathway in Ripk3-deficient cells under LPS injury. (D-E) Impact of AMPK on mitochondrial membrane potential. (F) The change of mPTP opening time. Mean ± SEM, ∗*p* < 0.05 vs. the Ctrl group; #*p* < 0.05 vs. the LPS group; &*p* < 0.05 vs. the LPS+AI group; %*p*< 0.05 vs. the LPS+cC group.

The mechanism by which Ripk3 triggers Drp1-mediated mitochondrial fission is still not fully understood. Multiple studies have asserted that the AMPK pathway is crucial in activating Drp1 phosphorylation at Ser637 and inhibits mitochondrial fission 37, 38. Therefore, our study examined whether the AMPK pathway was involved in Ripk3-induced mitochondrial fission. In order to address this question, AICAR (AI, 1mM), the AMPK pathway activator, was employed as the positive control, whereas compound C (cC, 10μM), the AMPK pathway inhibitor, was employed as the negative control, both in the presence of LPS. LPS inhibited AMPK pathway activation, evidenced by reduced phospho-AMPK expression, which was reversed by Ripk3 knockdown. **(Fig. 6A-B)**. In order to investigate the involvement of AMPK in the activation of mitochondrial fission, the level of phospho-Drp1 expression was measured. LPS inhibited the expression of phospho-Drp1 at Ser637. Nevertheless, the alterations were reversed as a result of Ripk3-knockdown, which had a resemblance to the administration of AICAR. In addition, the upregulation of phospho-Drp1 at Ser637 in Ripk3-knockdown cells was diminished by compound C **(Fig. 6A, C)**. The findings supported our hypothesis that Ripk3 controls mitochondrial fission related to Drp1 through the AMPK pathway. Moreover, the presence of LPS stress and compound C induced cellular oxidative stress, which was reduced by the knockdown of Ripk3 and the administration of AICAR. Meanwhile, we assessed the mitochondrial membrane potential. Compound C caused a reduction in mitochondrial membrane potential, which was subsequently restored by AICAR **(Fig. 6D-E)**. We additionally evaluated the rate of mPTP opening. The administration of LPS and compound C resulted in an elongated time of mPTP opening in WT cells, which was counteracted in Ripk3-deficient cells and treated with AICAR **(Fig. 6F)**. Overall, these findings indicate that Ripk3 knockdown lessens damage to the mitochondria via activating the phosphorylation of Drp1 at Ser637 through AMPK.

## Discussion

ALI, or its clinical manifestation, ARDS, is a significant health concern in developing countries, accounting for 30% of all deaths. ALI is characterized by noncardiogenic pulmonary edema with oxidative stress and inflammation affecting alveolar epithelial cells and pulmonary mesenchyme 39, 40. The aetiology of ARDS involves multiple factors such as ischemia, anoxia, severe infection, and trauma. These etiologies lead to lung injury through different mechanisms. In ARDS caused by viral pneumonia, viral invasion of lung cells activates the host's immune response. Viral nucleic acids are recognized as pathogen-associated molecular patterns (PAMPs) by intracellular pattern recognition receptors (PRRs), for example Toll-like receptors (TLRs) 41. This recognition process activates the Ripk3 signaling pathway and induces necroptosis in alveolar epithelial cells and pulmonary vascular endothelial cells. In ARDS caused by bacterial pneumonia, components such as LPS can stimulate inflammatory cells (such as macrophages) by releasing cytokines for example TNF-α 42, which can activate Ripk3 via the death receptor pathway when bound to cell surface receptors. In ARDS related to aspiration of gastric contents, acidic inhalant from the stomach will cause chemical damage to lung tissue after inhalation 43. This acidic substance directly harms alveolar epithelial cells and vascular endothelial cells, leading to cell damage and death. Meanwhile, damage-associated molecular patterns (DAMPs) are released, stimulating inflammatory cells in lung tissue, which in turn activate the Ripk3 signaling pathway, further damaging the structure and function of lung tissue and promoting the formation of ARDS. For sepsis-associated ARDS, toxins released by pathogens such as bacteria enter the blood circulation and cause systemic inflammatory responses 44. A large number of inflammatory factors such as TNF-α and IL-1β can activate Ripk3 through multiple signaling pathways, leading to necroptosis of lung cells, affecting the function of vascular endothelial cells, and promoting the formation of microthrombi, thus exacerbating pulmonary circulation disorders. After severe trauma, tissue damage releases large numbers of DAMPs, which trigger the body's immune response 45. At the same time, massive blood transfusion may cause transfusion-related acute lung injury (TRALI) 46. Inflammatory cell activation and increased oxidative stress due to trauma and blood transfusion may contribute to necroptosis of lung cells through activation of Ripk3, disrupting the alveolar-capillary barrier and ultimately leading to ARDS. The primary therapeutic approaches for ALI encompass antioxidant 47, anti-inflammatory 48, ventilation, and mesenchymal stem cell therapies 49; ARDS is a multifaceted condition that lacks targeted treatment options, although recent advancements in clinical care have led to better outcomes 50. Exploring new therapeutic methods and mechanisms is essential for developing more effective treatments for the devastating syndrome. Our findings contribute to this effort by identifying potential targets for novel ARDS therapies.

ARDS is characterized by dysfunction of the pulmonary microvascular endothelial barrier, a pathophysiological process significantly impacted by oxidative stress, which contributes to the breakdown of the barrier in PMVECs during the development of ARDS 51, 52. While a proper quantity of ROS is essential for facilitating signal transmission under physiological conditions, exposure to inflammatory substances can cause PMVECs to produce an excessive amount of ROS, leading to cytoskeleton contraction via myosin light chain (MLC) and the reduction of intercellular junction proteins. This results in the formation of gaps between cells, which brings increased permeability, fluid leakage, and the development of edema eventually 51. LPS is a primary causative factor of ALI, initially inducing mitochondrial injury, stimulating ROS overload, inhibiting oxidative phosphorylation, and causing elevated oxidative stress and damage to lung tissue 53, 54. The present research has determined Ripk3 to be a key regulator of LPS-induced ALI, providing compelling proof for the role of Drp1-induced mitochondrial fission and PMVEC necroptosis in the progression of ALI induced by LPS.

Mitochondria are recognized as organelles that function as a powerhouse, producing ATP to support various cellular processes, and also serve as a central hub for signaling and regulating various biological processes, such as metabolism, cell proliferation, and immune response. The respiratory chain of mitochondria is crucial for the transportation of electrons and the conversion of energy, and any disruption to this process can have an impact on energy metabolism and result in the disruption of tissue balance. Research has demonstrated that damaged mitochondria can disrupt the metabolic health of lung epithelial cells, resulting in various lung disorders including asthma, ARDS, lung cancer, and COPD 55, 56. The data provided strongly demonstrate the significance of preserving mitochondrial integrity to ensure tissue homeostasis and hinder the development of severe lung diseases. Mitochondria are targeted to trigger ALI under LPS challenge, since mitochondrial dysfunction has been shown to activate the NF-κB signaling pathway in lung tissue 57. Impaired regulation of mitochondrial quality is believed to be the primary cause of excessive ROS generation and oxidative stress in tubular cells during LPS-induced acute kidney injury 58. Septic cardiomyopathy is characterized by a decrease in mitophagy activity and a reduction in mitochondrial biogenesis. These factors contribute to a disturbance in cardiomyocyte energy metabolism and a failure in cardiac contractility 59. Consistent with these results, our investigation also observed impaired mitochondrial function and structural abnormalities in the mitochondria of lung tissue treated with LPS. Actually, extensive researches have been carried out to explore the role of mitochondrial dysfunction in ARDS. For example, ALI is characterized by metabolic changes regulated by mitochondria 60. Lung tissues treated with LPS have shown a decrease in ATP synthesis of mitochondria and an augment in ROS production of mitochondria 61. Interestingly, our current research has revealed significant changes in mitochondria morphology in lung tissue under LPS stress. In terms of mitochondrial injury, damage to mitochondria morphology is regarded as the initial sign, compared with mitochondrial function disorder. However, the hypothesis has not been verified in cases of ALI. Our research revealed that changes in mitochondrial morphology directly impact mitochondrial function in PMVECs treated with LPS. The discovery offers a fresh perspective on the molecular mechanisms involved in ALI induced by LPS.

Necroptosis is a newly discovered form of regulated cell death which is distinct from apoptosis, causing inflammation and damage to tissues. Ripk3 is a widely recognized protein kinase that promotes necroptosis, regardless of whether cell death is induced by interferons, TLR3, TLR4, or TNF superfamily death receptors. The level of Ripk3 expression plays a crucial role in deciding the specific model of cell death 62. Cells lacking Ripk3 are resistant to necroptosis. In acute lung injury, impaired alveolar epithelial and endothelial permeability involves multiple essential mechanisms, such as the activation of myosin light chain kinase (MLCK), RhoA and tyrosine kinases; increased calcium influx; and endothelial cell apoptosis 63, 64. It has been shown that the Nrf2-MafF/ARE signaling pathway inhibits apoptosis of endothelial cells under extensive inflammation and reduces oxidative damage in ALI/ARDS 65. In addition, mitoQ greatly reduces the high permeability of human pulmonary microvascular endothelial cells monolayer through regulating the expression of anti-apoptosis protein Bcl-2 and pro-apoptosis BAK, and decreases the activity of caspase 3 in endothelial cells, which are consistent with previous report that NFκB/NLRP3 pathway inhibits ROS and excessive autophagy in CSE-induced HUVEC injury 66. Recently, a correlated study has demonstrated that mitoQ inhibits apoptosis in lung tissues by activating the PI3K/Akt/GSK-3 β/mTOR pathway, protecting lung function 67. Unlike apoptosis, the understanding of the specific necroptosis mechanism is still limited in ARDS. The current study reveals that both apoptosis and necroptosis are stimulated by LPS, suggesting that necroptosis may have been overlooked as a causative element in ARDS. In addition, our findings further demonstrate that the Ripk3/Drp1 signaling pathway regulates LPS-induced necroptosis, and suppression of Ripk3 can delay the activation of necroptosis. However, there are still some unresolved matters that need to be addressed. Firstly, it remains uncertain whether necroptosis interacts with apoptosis in ARDS. Furthermore, it remains ambiguous whether they are controlled by a shared signaling pathway or upstream protein.

An association between Ripk3 upregulation and mPTP opening has been illustrated in several studies, which corroborates the necessity of mPTP for Ripk3-mediated necroptosis 68, 69. The necroptosis process following Ripk3 activation leads to an imbalance in intracellular calcium homeostasis. When the concentration of calcium ions in the mitochondrial matrix reaches a certain threshold, mPTP opening is triggered. At the same time, the necroptosis process is accompanied by an increase in ROS. In mitochondria, ROS can oxidatively modify key components of mPTP, such as adenine nucleotide translocator (ANT) and voltage-dependent anion channel (VDAC) proteins. This oxidative modification lowers the threshold for mPTP opening and drives the opening of the pore. In addition, it has been shown that MLKL may interact with proteins such as mitochondrial VDAC, which in turn alters the permeability of the mitochondrial membrane and affects the opening state of mPTP. During necroptosis, mitochondrial oxidative phosphorylation is impaired and ATP production is reduced, resulting in a decrease in mitochondrial membrane potential, which affects the stability of mPTP, making it easier to open. Our findings suggest that necroptosis is provoked by Ripk3 and executed by mPTP opening, answering the underlying mechanism of Ripk3 regulating mPTP opening, especially in the ALI setting.

In addition to mitochondrial fission, mitochondrial fusion also plays a crucial part in controlling the morphology and quantity of mitochondria, and these two activities usually maintain a constant balance. Drp1 is typically located in the cytoplasm and undergoes bidirectional movement between the cytoplasm and the outer surface of mitochondria 70. During periods of stress, including starvation and hypoxia, Drp1 can move from the cytoplasm to the mitochondria through phosphorylation 71. The molecules then congregate toward the outer membrane of the mitochondria, where they form oligomers and facilitate mitochondria fission 72. Our study confirms that Ripk3 enhances Drp1-mediated mitochondrial fission in acute lung injury generated by LPS. In addition, it has been verified that Ripk3 suppresses the process of mitochondrial fusion in septic cardiomyopathy 73. Further research is necessary to ascertain the impact of Ripk3 on mitochondrial fusion in lung injury linked to inflammation.

Drp1-mediated mitochondrial fission is controlled through various post-translational modifications, including SUMOylation, ubiquitination, and phosphorylation 74. Phosphorylation is known to regulate the activity of Drp1. Phosphorylation at Ser637 inhibits mitochondrial fission, which ultimately reduces necroptosis 75. AMPK, a key detector of energy strain, can be activated (predominantly phosphorylation at Thr172) by various mitochondrial stimuli and promptly affects mitochondrial fission 76, 77. Thus, AMPK is an essential focus for the management of ALI. There is ongoing debate regarding the impact of AMPK on the process of mitochondrial fission. A recent research has found that AMPK stimulates mitochondrial fission through upregulating Mff 78, while some research has indicated that AMPK hinders mitochondrial fission by regulating Drp1 expression in aortic endothelial cells 79. Our data showed that the inhibition of Ripk3 via a lentivirus system activated the AMPK pathway by causing phosphorylation at Thr172 in PMVECs. AMPK activation promotes the phosphorylation of Drp1 (Ser637) and also prevents Drp1-mediated mitochondrial fission. Our research uncovered the role of the AMPK/Drp1/mitochondrial fission axis in controlling LPS-triggered necroptosis in ALI.

It's worth noting that the environment of cell culture *in vitro* is not exactly the same as the environment of the complex cellular population *in vivo*, and *in vitro* culture lacks regulation by the nervous and endocrine systems. Lacking these regulatory controls, the metabolism of *in vitro* cells is more constant than that of *in vivo* cells, but this is not a true representation of the tissue from which the cells originate. In addition, compared with *in vivo* cells, cells cultured *in vitro*, especially those that have been repeatedly passed on and cultured for a long time, have undergone certain changes in morphology and function, and may undergo the phenomenon of dedifferentiation and screening, resulting in the loss of some biological properties of the original cells. Moreover, cells cultured *in vitro* lack interaction with other different types of cells, which makes the physiological response ability of cells become single and has certain limitations.

**Figure 7:**
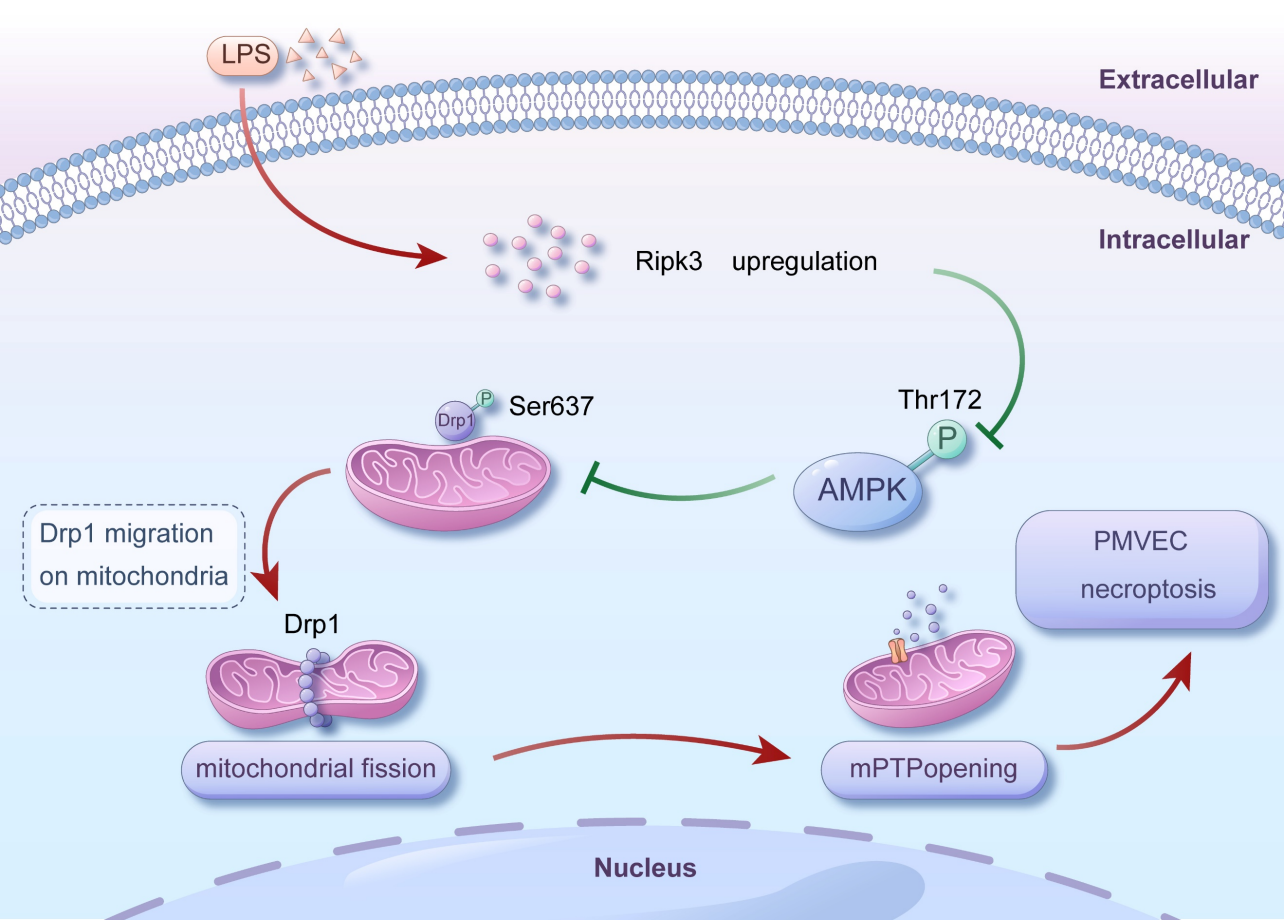
** Mechanism diagram for the role of Ripk3-AMPK-Drp1-mPTP in LPS-mediated acute lung injury via initiating necroptosis.** LPS injury caused upregulation of Ripk3, which resulted in a reduction in the phosphorylation of AMPK. Inactivating AMPK hindered the phosphorylation of Drp1 at Ser637, which triggered fatal mitochondrial fission. Excessive fission caused the opening of mPTP, ultimately leading to the necroptosis of PMVECs. Nevertheless, the recovery of AMPK activity could halt the excessive fission, offering prosurvival effects for the lung in the presence of LPS injury. Our findings revealed a novel role for Ripk3 in LPS-induced acute lung injury through regulating Drp1-mediated mitochondrial fission via the AMPK signaling pathway. These results suggest that targeting Ripk3 could be a promising therapeutic approach for treating acute lung injury in clinical practice.

Given the above, our studies have revealed a new role of Ripk3 in ALI induced by LPS. Specifically, Ripk3 controls mitochondrial fission mediated by Drp1 through the AMPK signaling pathway. The buildup of excessive mitochondrial fragments results in function disorder of mitochondria and the opening of mPTP, ultimately triggering necroptosis in PMVECs and impairing migration** (Fig. 7)**. These discoveries provide insight into the therapeutic potential of targeting Ripk3 as a remedy for ALI. By inhibiting the activity of Ripk3, necroptosis can be blocked, thereby attenuating lung injury. At present, some inhibitors targeting Ripk3 have been developed and have shown certain efficacy in animal experiments. For example, a newly developed Ripk3 inhibitor, UH15-38, can effectively and selectively block necroptosis of alveolar epithelial cells triggered by influenza A virus *in vivo*, improve lung inflammation, and prevent death after infection, which provides a certain reference for drug development of ALI 80. Meanwhile, Ripk3 inhibitors have the potential to be used in combination with other therapeutic agents for ALI, such as anti-inflammatory drugs and antioxidant drugs, with a view to playing a better role in the treatment of ALI.

Initiation of the Ripk3 signaling pathway promotes fibroblast activation and proliferation in lung tissue, increases extracellular matrix deposition and influences extracellular matrix remodeling, providing a cellular basis for the development of pulmonary fibrosis 81. Appropriate regulation of Ripk3 during ALI regression helps to reduce the infiltration of inflammatory cells into lung tissue. By inhibiting Ripk3, its downstream necroptosis signaling pathway is blocked, which decreases inflammatory mediators released by inflammatory cells (such as neutrophils and macrophages) due to necroptosis, and at the same time alleviates the occurrence of necroptosis in alveolar epithelial cells, which contributes to maintaining the integrity of the alveolar epithelial cell-to-cell junctions, and promotes the recovery of alveolar function.

Given that the function of mitochondria is controlled by various activities such as mitophagy, mitochondrial fusion and fission, the precise molecular mechanism of acute lung injury requires more clarification.

## Conclusion

In summary, acute lung injury is correlated with elevated Ripk3 expression, resulting in inhibition of AMPK. The reduction in AMPK subsequently hinders the phosphorylation of Drp1 (Ser637), leading to excessive stimulation of mitochondrial fission, increasing the opening of mPTP and resulting in cellular necroptosis in acute lung injury. Therefore, by genetically eliminating Ripk3, the AMPK/Drp1-mitochondrial fission pathway can be activated to prevent mPTP opening and PMVEC necroptosis. As a result, acute lung injury can be finally avoided. The outcomes of our study offer a hopeful target for the management of ALI.
